# Miniaturization for ultrathin metamaterial perfect absorber in the VHF band

**DOI:** 10.1038/srep45151

**Published:** 2017-03-22

**Authors:** Bui Xuan Khuyen, Bui Son Tung, Young Joon Yoo, Young Ju Kim, Ki Won Kim, Liang-Yao Chen, Vu Dinh Lam, YoungPak Lee

**Affiliations:** 1 Department of Physics and RINS, Hanyang University, Seoul, South Korea; 2 Department of Display Information, Sunmoon University, Asan, South Korea; 3 Fudan University, Shanghai, China; 4 Institute of Materials Science, Vietnam Academy of Science and Technology, Hanoi, Vietnam

## Abstract

An efficient resolution for ultrathin metamaterial perfect absorber (MPA) is proposed and demonstrated in the VHF radio band (30–300 MHz). By adjusting the lumped capacitors and the through vertical interconnects, the absorber is miniaturized to be only λ/816 and λ/84 for its thickness and periodicity with respect to the operating wavelength (at 102 MHz), respectively. The detailed simulation and calculation show that the MPA can maintain an absorption rate over 90% in a certain range of incident angle and with a wide variation of capacitance. Additionally, we utilized the advantages of the initial single-band structure to realize a nearly perfect dual-band absorber in the same range. The results were confirmed by both simulation and experiment at oblique incidence angles up to 50°. Our work is expected to contribute to the actualization of future metamaterial-based devices working at radio frequency.

In recent years metamaterials (MMs) with a sub-wavelength-scale unit cell have become extremely attractive in the field of material science. MMs are well-known as artificial structures (so-called meta-atoms) designed to interact with the electric and the magnetic components of electromagnetic (EM) waves in a different way from natural interactions with normal atoms. Researches on MMs have witnessed a prompt growth and uncovered exciting new effects and technologies such as: negative refractive indices^1^,^2^,^3^,^4^, invisibility cloaking^5^, and perfect lenses^6^,^7^. Particularly, the unique ability to achieve unity absorption with high efficiency was firstly realized by Landy *et al*. in 2008 by using the concept of MM perfect absorber (MPA)^8^. Essentially, the real and the imaginary parts of permittivity *ε(ω*) and permeability *μ(ω*) can be controlled independently in order to obtain an effective impedance that perfectly matches the impedance of free space, and the imaginary part of refractive index can be extremely enhanced. Consequently, a transmitted EM wave with zero reflection can be absorbed perfectly and dissipated by dielectric loss and strong resonance. To date, MPAs have been thoroughly studied from the microwave to optical range^9^,^10^,^11^,^12^,^13^,^14^ and are promising for a wide range of application areas such as bolometers^15^, thermal images^16^,^17^, solar cells^18^, sensors^19^, and optical switches^20^.

One currently-expanding field is the actualization for MPAs working at very low frequencies due to the rapid growth of telecommunication devices^21^,^22^,^23^,^24^,^25^,^26^,^27^,^28^,^29^. Hence, many effective resolutions have been proposed for scaled-down MPA structures with small unit cell. For instance, Costa *et al*. suggested a thin MPA with a periodicity of *λ*/2 and a thickness of *λ*/44 for sub-GHz wireless systems^27^. Yoo *et al*., then, successfully demonstrated a flexible MPA, which was miniaturized to be *λ*/30 and *λ*/62 at 400 MHz for the unit-cell width and thickness, respectively, based on a snake-shape structure^28^. Lately, in an UHF (ultrahigh-frequency) band (910 MHz), another MPA with lumped resistors, whose lattice constant and thickness are miniaturized to be *λ*/17 and *λ*/15, respectively, has been introduced for RFID (radio-frequency identification) systems^29^. These examples indicate that it is very difficult to obtain simultaneously thin and small MPAs. Therefore, to build meta-instruments that work in the radio region, the efficient miniaturization of MPAs is still a challenge that must be overcome. For that reason, we suggest a new class of ultrathin MPAs, where not only the thickness is extremely thin (*λ*/816) but the periodicity is also small (*λ*/84) with respect to the absorption wavelength in the very-high-frequency (VHF) band (30–300 MHz). By containing lumped capacitors in a compact structure with through interconnects, the incoming energy of EM wave is consumed at very low frequency. A simplified equivalent circuit model is used to estimate the operative frequency in the defined band. The practical performance for our MPA is also verified via the variation of incident angle and polarization of EM wave. Furthermore, we realize an ultrathin dual-band MPA in the same frequency range.

## Design, Results and Discussion

**Figure 1:**
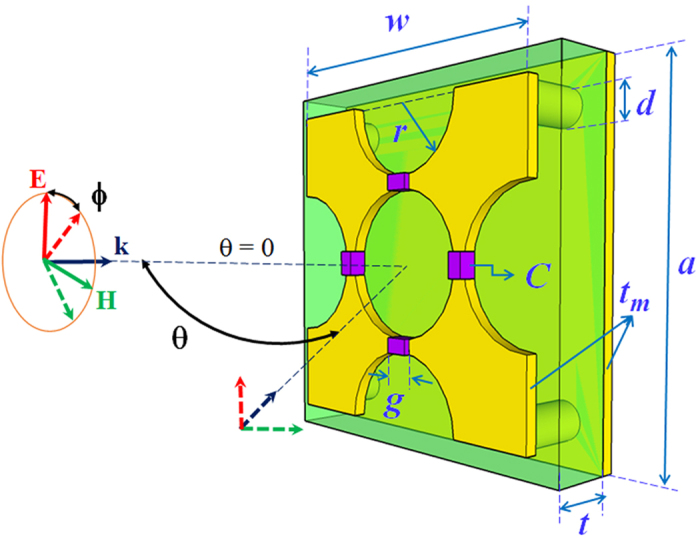
3-dimensional periodic structure of the unit cell of proposed MPA with the polarization of EM wave.

The 3-dimensional schematic for a single unit cell (periodicity of *a*) of an ultrathin MPA involving three layers (metal-dielectric-metal) is shown in Fig. 1. The top meta-surface is a square (width of *w*) with a circular cutout in the center and four semi-circular cutouts in the center of each side (radius of *r*). Four corners of the top meta-surface are connected to the bottom continuous metallic ground plate by four through interconnects (cylindrical wires with diameter of *d*) through a dielectric spacer. The air gaps (width of *g*) are made at the knots of the top meta-surface, where four capacitors (capacitance of *C* = 150 pF) are embedded. The optimized geometrical parameters of proposed MPA are *a* = 35, *w *= 33, *r* = 7.8, *g* = 1.6, and *d *= 2.5 mm. The dielectric spacer is chosen as FR-4 (thickness *t *= 3.6 mm) with a dielectric constant of 4.3 and a loss tangent of 0.03. The metal for the top and the bottom layers (thickness *t*_*m*_* *= 0.036 mm) and the through vertical interconnects are selected as copper with an electric conductivity of *σ *= 5.8 × 10^7^ S/m. The key idea of our design is a combination of lumped capacitors on the optimized meta-surface and copper interconnects in order to achieve strong resonance and perfect impedance matching simultaneously at very low frequencies. These unique advantages are described in Fig. S1 of Supplementary, where the important roles of through interconnects and capacitors on the ultrathin MPA design are recognized.

**Figure 2:**
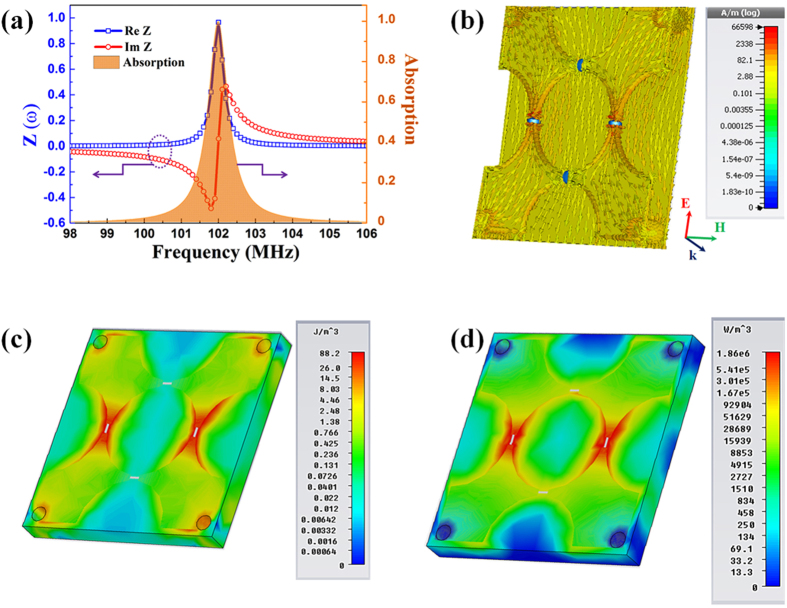
(**a**) Simulated effective impedance and absorption spectrum of the proposed MPA. 3-dimensional distributions for (**b**) induced surface currents, (**c**) magnetic energy, and (**d**) power loss at the resonant frequency.

As shown in Fig. 2(a), a nearly-perfect absorption peak (absorption of 99.8%) is obtained at 102 MHz (as the orange area). Interestingly, the ratios between thicknesses *t* and lattice constant *a* of MPA to the operation wavelength λ at 102 MHz are optimized to be only: *t*/*λ *= 1/816 and *a*/*λ *= 1/84. These values are the smallest among the currently-published structures^21^,^22^,^23^,^24^,^25^,^26^,^27^,^28^,^29^. The nature of perfect absorption can be explained by the impedance matching theory^30^. At the absorption frequency, by controlling the electric permittivity *ε(ω*) and the magnetic permeability *μ(ω*), the effective impedance of the MPA is perfectly matched with free space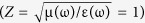. Based on the reflection and the transmission parameters, the effective impedance can also be given by ref.^31^:


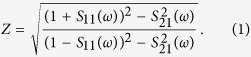


Both extracted real and imaginary parts of the relative impedance are plotted in Fig. 2(a). It is clearly observed that the effective impedance of MPA has an imaginary part of zero and a real part of approximately 1.0 at 102 MHz. Consequently, there is no reflected wave between the MPA and the surrounding environment.

In association with the perfectly-matched impedance requirement, the effective energy consumption of incoming EM wave is produced by intrinsic resonance inside the MPA. This phenomenon can be visualized by the simulated surface-current, magnetic-energy and power-loss-density distributions at the absorption frequency in Fig. 2(b–d). The 3-dimensional view in Fig. 2(b) shows the induced currents flowing in the structure at 102 MHz. The induced surface currents, which are caused by excitation of the EM field, accumulate in a bottom-up direction on the front patterned-surface and a top-down direction in the back metallic layer. With the interconnection of the cylindrical copper wires at each corner, the induced loop currents are formed within the MPA and perpendicular to the direction of external magnetic field (**H**). These loop currents prove that a magnetic resonance is produced as expected in the common MPAs. In other words, the perfect absorption originates from the magnetic resonance where the perfect impedance matching arises simultaneously at 102 MHz for our MPA. It is noteworthy that the induced currents have high magnitudes at the knots of meta-surface and on the corresponding areas of the ground plate, due to the coupling between these layers. This phenomenon makes the magnitude of magnetic energy strongly enhanced in the specific areas as shown in Fig. 2(c). The aforementioned distribution of loop currents also cause the induced power loss of EM wave [Fig. 2(d)]. Evidently, the energy of the incoming EM wave is consumed inside the absorber. The power loss (dielectric loss) is mostly concentrated around the left-right capacitor locations along the external electric-field (**E)** direction. In short, inside the ultrathin structure, the strong magnetic resonance and the perfect impedance matching play significant roles in achieving the perfect absorption at 102 MHz.

**Figure 3:**
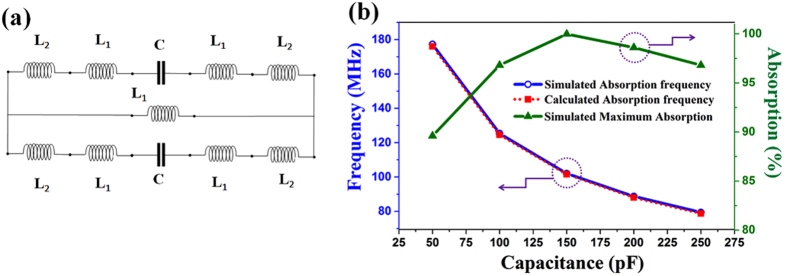
(**a**) Equivalent circuit of the discussed MPA. (**b**) Simulated and calculated absorption frequencies according to the value of lumped capacitor. Red-square and blue-circle symbols represent the calculated and the simulated results, respectively. Green-triangle symbols mark the simulated absorption.

To theoretically evaluate the absorption frequency (the magnetic resonance) in addition to the simulation, we provide the equivalent inductor-capacitor (LC) circuit model as in Fig. 3(a) 23,32,33. *L*_*1*_ and *L*_*2*_ are the effective inductances of the top quarter of meta-surface and the via interconnect located at each quadrant of the unit cell, respectively. *L*_*1*_ and *L*_*2*_ are optimized to be 7.23 and 0.949 nH, respectively. In this model, *C *= 150 pF is the capacitance of lumped element and the resistor element is ignored for simplicity (it is small and does not affect the frequency position). The total circuit impedance in Fig. 3(a) is given by:


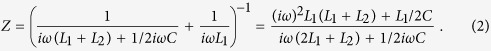


Consequently, the magnetic resonant frequency can be simply derived by the condition of Im(Z)* *= 0 from Eq. (2):


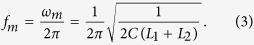


Equation (3) denotes that the absorption frequency is inversely proportional to the square root of effective inductances and lumped capacitance. It is noteworthy that, if we apply the conventional MPA design (without lumped capacitors and through vertical interconnects), the inductance *L*_*2*_ would be removed and the capacitor *C* would be replaced by an intrinsic effective capacitor *C*_*m*_ caused by the middle dielectric layer. Owing to the lack of *L*_*2*_ and the small *C*_*m*_ (we estimated that its value was less than 5 pF in our structure), the operational frequency of this traditionally-designed MPA shifts towards a much higher frequency in the GHz range [see Fig. S1(a) in Supplementary]. Therefore, by adding the large capacitor and by the contribution of *L*_*2*_, the low absorption frequency is efficiently achieved in the VHF range even with the ultra-small unit cell. The preeminent advantage of structure is displayed in Fig. 3(b), where the dependences of calculated and simulated absorption frequencies on capacitance *C* are plotted. When *C* is increased from 50 to 250 pF, the absorption peak is red-shifted from 177.2 to 79.4 MHz while the absorption rate is maintained at or above 90%. It can be seen clearly that the theoretical model is highly consistent with the simulated results. Furthermore, the result suggests that our ultrathin MPA might be useful for tunable applications in the VHF band when a variable capacitor is incorporated instead of the normal one.

**Figure 4:**
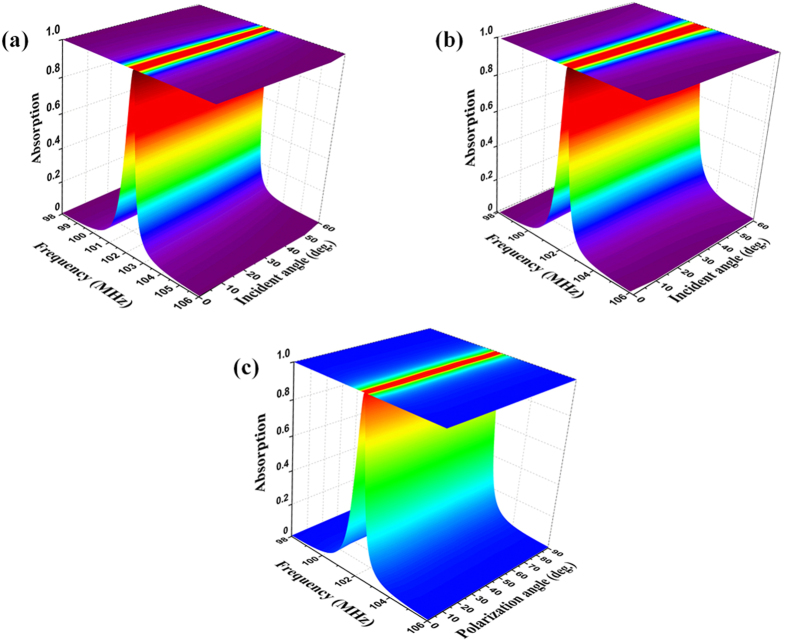
Simulated absorption spectra of the ultrathin MPA according to the incident angle of EM wave for the (**a**) TE and (**b**) TM polarizations. (**c**) Dependence of absorption on the polarization angle of normal EM wave.

In Fig. 4, the dependence of absorption on the oblique angle of incidence (*θ*) and the polarization (ϕ) was investigated to evaluate the operational capability. Remarkably, as presented in Fig. 4(a,b), the absorption of 99.8% at 102 MHz when *θ *= 0 falls only slightly to 90% when *θ *= 55° for both transverse-electric (TE) and transverse-magnetic (TM) polarizations. In other words, the designed MPA conserves well the impedance-matching condition for a wide range of incident angle. Furthermore, by exploiting the symmetric structure, the ultrathin MPA exhibits the polarization-insensitive behavior [as shown in Fig. 4(c)]. The absorption peaks (absorption over 99.8% at 102 MHz) are nearly unchanged for the overall polarization angles from 0 to 90° for the normal-incident wave. These results can be also applied to many real applications, which demand a wide range for oblique incidence and the independent polarization of EM waves.

**Figure 5:**
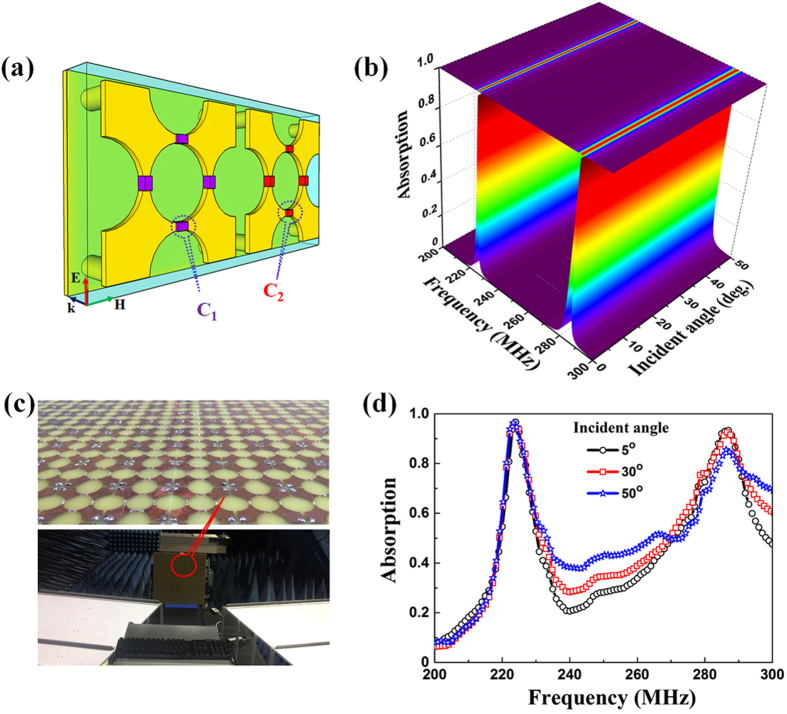
(**a**) Schematic of the proposed dual-band MPA structure with the polarization of EM field. (**b**) Simulated absorption spectra according to the incident angle of EM wave. (**c**) (Bottom) Illustrated arrangement for the experimental configuration and (top) magnification of the fabricated sample. (**d**) Measured absorption spectra of the dual-band MPA according to the oblique incidence angle.

In order to realize the advantages of our design, we propose a simple method to create dual-band perfect absorption. According to the aforementioned analysis (Fig. 3), the strong magnetic resonance and the impedance matching are maintained well in a wide range of lumped capacitance. Estimating from the LC equivalent circuit, we can select the capacitance values corresponding to the expected frequency regions. For the super cell in Fig. 5(a), two capacitors (*C*_1_* *= 40 pF and *C*_2_* *= 25 pF) are integrated into two adjacent unit cells of the initial single-band design (*t* = 2.6 mm and the other parameters are fixed) to excite two independent magnetic resonances. As a consequence, the dual-band perfect absorption is recognized in the same region as shown in Fig. 5(b). In the normal incidence, two absorption peaks at 224.9 MHz (corresponding to *C*_1_* *= 40 pF) and 284.2 MHz (corresponding to *C*_2_* *= 25 pF) are clearly observed with absorption of 99.9% and 98.5%, respectively. The detailed energy consumption is described in Fig. S2 in Supplementary Information. As the incident angle is increased to be 50°, the absorption is still over 99.3 and 90% for the respective frequencies. Principally, the dual-band MPA is very thin and has a short periodicity with respect to the operating wavelengths (*t*/*λ* = 1/513 and 1/406; *a*/λ* *= 1/19 and 1/15 corresponding to the lower and the higher frequency).

The evolution of measured absorption data [Fig. 5(d)] of the fabricated sample is in good agreement with the simulated spectra in Fig. 5(b). When *θ *= 5°, the measured absorption reaches 96% (at 224.2 MHz) and 93.3% (at 285.6 MHz). For an incident angle of 50°, the absorptions are detected as 94.8 and 87% for the lower and the higher frequency, respectively. The small deviations between measured and simulated absorption spectra can be largely due to the scattering from imperfections in the fabricated sample and the scattering between the two horn antennas caused by their relatively-large aperture^34^. In spite of these discrepancies, the dual-band MPA with high absorption is realized for a wide range of the incident angle.

## Conclusions

The perfect absorption features for a new type of ultrathin MPA are studied by experiment, simulation and calculation in the VHF region. By exploiting the magnetic resonance in the special structure, the unit cell of absorber is efficiently miniaturized to be 35 ×  35 × 3.6 mm^3^ (corresponding to periodicity of 0.012λ and thickness of 0.0012λ) at 102 MHz. The operation of designed MPA (absorption over 90%) is entirely stable for a wide range of incident angle (up to 55°) for the TE and TM polarizations. The simulation also proves that the absorption is polarization-insensitive at the normal incidence of EM waves. Particularly, by tuning the lumped capacitance of the initial MPA structure, we effectively realized dual-band perfect absorption in the same frequency region. Both experiment and simulation confirm that two absorption peaks (absorption about 90%) are well maintained for an incident angle up to 50°. Thus, the benefits of our optimized structure can be applied to future meta-devices in the radio band.

## Method Summary

### Simulations

Our simulations were conducted on a commercial software package, CST Microwave Studio^35^. The absorption can be calculated as 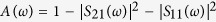, where 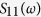 and 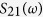 are the reflection and the transmission parameters, respectively. Because the back layer is a continuous metallic plane, 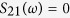, therefore, the absorption is expressed as 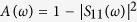.

### Fabrication

Figure 5(c) presents the sample of dual-band absorber. The dual-band MPA is fabricated with a size of 800 mm × 800 mm × 2.6 mm (approximately 23 × 11 unit cells), which is large enough to adequately include the main beam of transmitting antenna. The meta-surface and the through vertical interconnects are precisely fabricated by the printed-circuit-board technique (photolithography and high-speed steel-drilling processes). The real capacitors are chosen as chip multilayer ceramic capacitors (MLCC-SMD/SMT 40 and 25 pF) that are suitable for the operation at microwave frequencies.

### Measurement

The experimental configuration of measurement is arranged in Fig. 5(c). The reflection spectra were measured by using a Hewlett-Packard E8362B network analyzer (operational frequencies from 200 MHz to 20 GHz). In order to allow the EM waves to radiate sufficiently and to minimize the near-field effects, the distance between antennas to sample is kept at 6.8 m (approximately 6 times bigger than the absorption wavelength), which is farther than the far-field of antenna in the microwave anechoic chamber. The aperture of two horn antennas and the distance from sample to the middle point of two horn antennas were finely established for incident angles of EM waves from 5° to 50°.

## Additional Information

**How to cite this article:** Khuyen, B. X. *et al*. Miniaturization for ultrathin metamaterial perfect absorber in the VHF band. *Sci. Rep.* **7**, 45151; doi: 10.1038/srep45151 (2017).

**Publisher's note:** Springer Nature remains neutral with regard to jurisdictional claims in published maps and institutional affiliations.

## Supplementary Material

Supplementary Information
