# Supplementary material for: Miniaturization for ultrathin metamaterial perfect absorber in the VHF band
Source: Sci Rep. 2017 Mar 22;7:45151. doi: 10.1038/srep45151 (PMC5361105; doi:10.1038/srep45151)
Supplement: Supplementary Information [file srep45151-s1.pdf]

Supplementary Information for "Miniaturization for ultrathin metamaterial perfect absorber in the VHF band"

Bui Xuan Khuyen, Bui Son Tung, Young Joon Yoo, Young Ju Kim, Ki Won Kim,
Liang-Yao Chen, Vu Dinh Lam, and YoungPak Lee

Here, in this Supplementary Information, we present details on the advantages of the proposed design for ultrathin metamaterial perfect absorbers (MPAs) at low frequency and details on the electromagnetic-wave absorption mechanism of the dual-band MPA.

I. Optimization for an ultrathin metamaterial absorber

To evaluate the effects of the key constituent elements on the operation of an ultrathin MPA at the fundamental absorption frequency, we perform the simulations for three different structures with the same geometrical parameters. Figure S1(a) shows the typical spectra of the common metamaterial absorber (MA) including only the meta-surface as the top layer (without capacitors and through vertical interconnects). The maximum absorption reaches only 33.5% [as the orange area] at 4.0 GHz [where Re(Z) = 10 and Im (Z) = 0]. In the corresponding LC-circuit theory, to achieve high absorption at lower frequency, the total effective inductance (L_{eff}) and capacitance (C_{eff}) should be optimized to be larger ($f = 1/2\pi\sqrt{L_{eff}C_{eff}}$). To solve this problem for the small MA structure, firstly, the extra effective inductance of the copper interconnects, which are connected throughout the meta-surface to the ground metallic layer at four corners (without capacitors), are sufficiently added in the total effective inductance. The efficiency for this method can be clearly witnessed in the Fig. S1(b), where the absorption peak shifts from 4.0 [in Fig. S1(a)] to 2.1 GHz. It is noteworthy that the absorption can be improved to be 81% by the better impedance matching [Re(Z) = 2.5, Im (Z) = 0 at 2.1 GHz]. Secondly, the low frequency absorption can also be obtained by the lumped capacitors integrated at the specific locations

on the MA pattern. This solution is applied to our design and presented in Fig. S1(c), where four capacitors (150 pF) are incorporated at each knot of the meta-surface (without through interconnects), the absorption is improved up to 84% and the absorption peak is efficiently reduced to be at 1.4 GHz. It can be concluded that, for an ultrathin MPA, the through vertical interconnects and capacitors play the dominant roles in maintaining the high absorption amplitude and matching perfectly the impedance simultaneously in low frequency range.

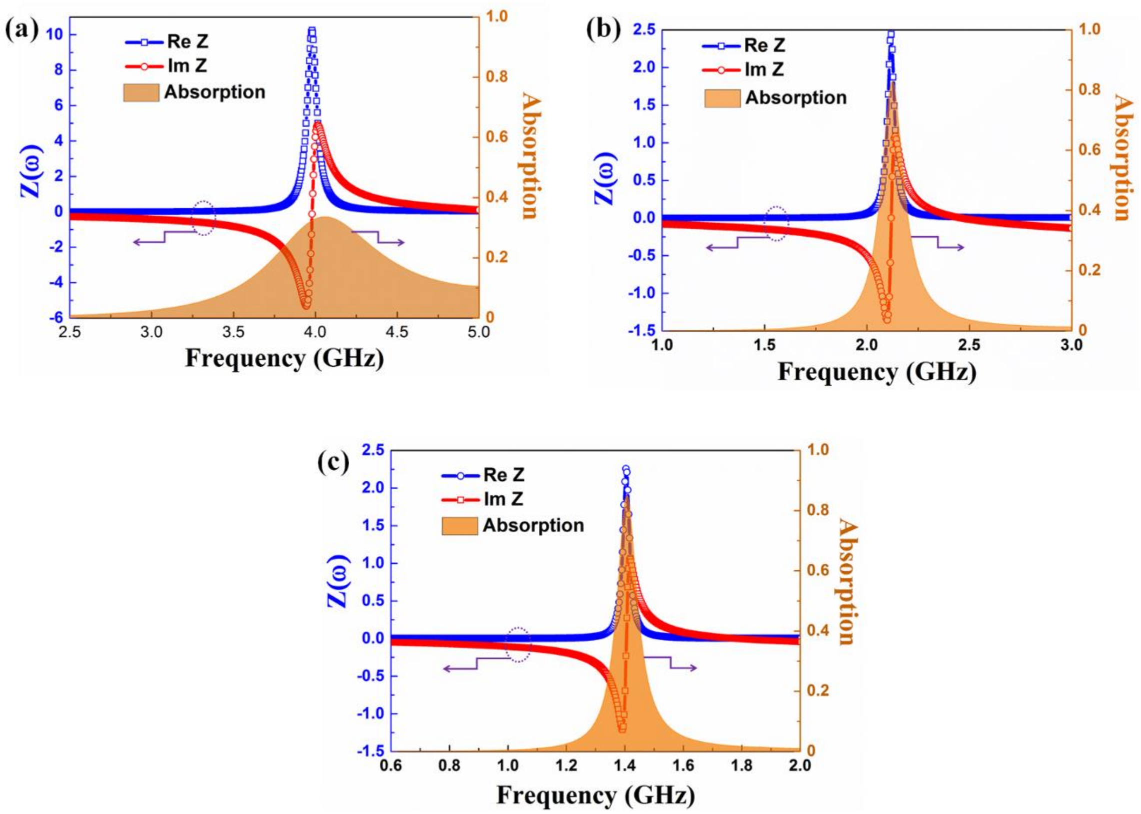

Figure S1. Calculated effective impedance and simulated absorption spectrum of the same unit cell, which contains: (a) only meta-surface (without capacitors and through vertical interconnects); (b) meta-surface incorporating with through vertical interconnects at four corners (without capacitors); (c) meta-surface integrating with the lumped capacitors at four knots (without through vertical interconnects).

II. Energy consumption mechanism of the dual-band MPA

The mechanism is better to be explained with Fig. S2 below.

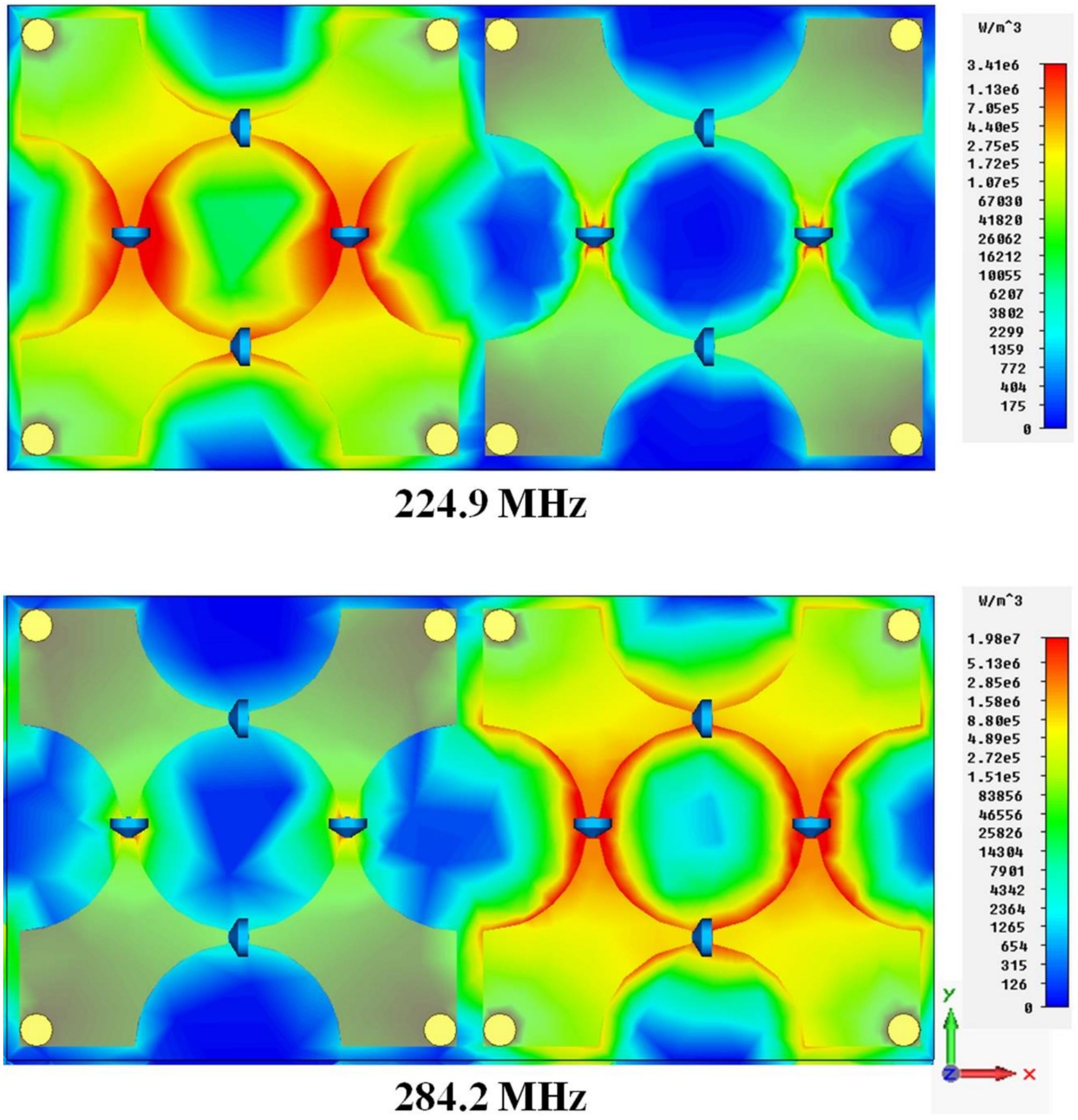

Figure S2. Distribution of the power loss density of MPA super cell of two unit cells at low and high frequencies. By exploiting the strong magnetic resonance, the energy of incident electromagnetic wave is perfectly consumed inside the MPA. The power of loss density is highly enhanced on the single unit-cell integrating with 40-pF capacitors in left unit cell (for an absorption peak at 224.9 MHz) and the other one with 25-pF capacitors in right unit cell (for an absorption peak at 284.2 MHz).
